# Introspective Projection: Prototypical Representations of Policing in the Service of Rule of Law

**DOI:** 10.1007/s12124-021-09632-w

**Published:** 2021-08-16

**Authors:** Gordon Sammut, Rebekah Mifsud, Noellie Brockdorff

**Affiliations:** 1 Department of Criminology, University of Malta, Msida, Malta; 2 Department of Cognitive Science, University of Malta, Msida, Malta

**Keywords:** Introspection, Projection, Social identity, Social representations, Policing, Rule of law, Democracy

## Abstract

Mass protests that have taken place over the past decade in various Western democracies have called into question the role of police in society, as officers have employed measures to contain rallies protesting for or against various issues. A number of these protests have resorted to violent means, resisting the police or protesting directly against their role and methods. The present study sought to investigate the prototypical representations of the police that lay citizens use to forge or desist identification with police officers. Social identification enables citizens to consider the police as ingroup members, facilitating respect for their authority. Conversely, identifying the police as outgroup precipitates resistance. The study involved 41 in-depth interviews carried out with citizens of Malta between May and June 2020. Thematic Networks Analysis revealed various points of consensus as well as a number of controversial themes. In particular, respondents demonstrated sceptical attitudes regarding policing on the beat for fear of overfamiliarity, rooted in introspective attributions projected at the police as merely human. Moreover, respondents expressed support for technological innovations that overcome natural psychological tendencies. The findings of this study suggest that seeking increasing trust in the police may be a red herring for policymakers. Rather, efforts should be directed at developing inter-objective systems, (e.g. body-cams), that overcome individual psychological propensities.

## Introduction

Over the past decade, Western democracies have witnessed a resurgence of crowd protests in pursuit of social change (Sammut & Bauer, 2021). From the Occupy Wall Street movement in New York, to the anti-austerity marches across Europe, the *giletes jaunes* in France, the Brexit protests in Britain, the Catalan protests in Barcelona, as well as anti-Covid-19 measures rallies in Germany, along with various others, crowds have routinely gathered in Western democratic nations to stage protests against various government policies. More recently, parts of the United States have been crippled by the Black Lives Matter protests targeting policing practices. Similarly, in France, crowds have protested the introduction of measures intended to protect the identities of police officers from the malicious slandering by lay citizens over the media. At the heart of these and other mass movements is the democratic challenge of institutional functioning in contexts of diversity and disagreement. Unlike despotic regimes, democratic societies champion human rights that include freedom of expression (Moghaddam, 2016). These rights empower citizens to publicly take issue with what they consider as untoward, including government policy mandated by the democratic will of citizens expressed in free and open elections. However, this does not mean that democratic freedoms obtain consensus in controversial matters. Quite the contrary, democratic societies allow for disagreement to be openly expressed and for public opinion to polarise in advancing one perspective over another. Political action, in turn, may lead down a route where, in the words of the European Commissioner for home affairs Ylva Johannson: No one will be satisfied (EU Observer, 2020).

This is where, we argue, social psychological mechanisms of identification come into play. To the extent that institutional authorities, such as the police, are deemed one of us (e.g. helping us out when we’re collectively fighting an unknown and invisible enemy – *Coronavirus*), they stand to be lauded as heroes. On the other hand, to the extent that they are perceived as ‘other’ (e.g. sniping to fine hapless citizens who forgot to don their Covid19-proof mask), they are an interfering authority limiting our ability to exercise our hard-won democratic rights and freedoms. The same can be said for other authorities exercising particular duties in contemporary democratic societies (e.g. parliament, the courts, medical professions, religious organisations, etc.), as well as for any other issue (e.g. immigration, policing, corruption, human rights, etc.) that precipitates collective protests and civil unrest. We argue that understanding the identity concerns underlying social movements serves in managing public expectations towards ensuring maintenance of social order and the neutralisation of conflict spirals between opposed coalitions-for-action (Buhagiar & Sammut, 2020; Sammut et al., 2015a, 2015b).

In this paper, we adopt a social representations approach (Sammut & Howarth, 2014; Sammut et al., 2015a, 2015b) to investigate social identity processes underlining support or resistance towards the Police Corps in Malta. Like other countries, Malta has seen its share of mass protesting in recent years. Following the assassination of investigative journalist Daphne Caruana Galizia, mass protests against government corruption led to the resignation of the prime minister along with a number of other cabinet ministers. Daphne Caruana Galizia’s murder remains controversial also in view of the fact that she had reportedly turned down police protection due to her distrust of Malta’s governing authorities, including the police. In this paper, we argue that a fuller understanding of underlying social identification processes can help the promotion of a democratically responsible citizenry (Moghaddam, 2016). Our study sheds light on both the controversy and the consensus that inheres in the social representation of policing in Malta, which is systemically shared across a diversified citizenry subject to institutionalised law enforcement. We proceed to outline the theoretical framework that guided our inquiry before we review methodological details and present our findings. We conclude with a discussion concerning institutional responsibilities relative to a diversified democratic citizenry, with special reference to the police. The findings of this paper highlight the implications of prototypical representational work in understanding interpersonal behaviour. We argue that social identification involves an element of introspective projection and that inter-objective systems (Sammut et al., 2010) may overcome limitations attributed to human fallibility.

## Identity and Prototypical Representations

Social Identity Theory [SIT] (Tajfel & Turner, 1979) posits that an individual’s identity contains a reference to the social groups the individual is a member of. In this way, social identity constitutes that element of identity which describes who one is – for others. One could personally subscribe to particular social groups, either privately (e.g. Freemasons) or publicly (e.g. Arsenal Football Club supporters). Moreover, individuals could be assigned to particular social groups by others and not necessarily out of their own personal sense of identification (e.g. *African*-American, see Howarth et al., 2014). In any case, our social identity positions us in the public domain relative to others, as individuals sharing some essence in common with similar others. The social representation of these core essences helps confer status to certain social identities whilst deprecating others, depending on the social value assigned to the category. For instance, Buhagiar et al. (2018) show how the attribute ‘Muslim’ carries negative connotations across Europe and is conferred on members of Arab countries regardless of their diversity. Conversely, other social representations of essential features associated with other social groups convey social prestige (e.g. scholars as intellectually superior, see Sowell, 2012). The crucial point here is that regardless of the extent to which one personally identifies with the categories they are assigned to, one’s social identity serves to position an individual with some and, simultaneously, against some others. This is, in essence, how stereotypes function in the social domain (Sammut, 2015).

Hogg (2001) proposed an extension of SIT to account for the phenomenon of leadership. According to Hogg, the notion that leaders command some extraordinary characteristics that enables their rise to power is mistaken. Rather, their ascent is a direct function of the actions of their followers. Specifically, leadership is a function of three interlinked processes. Firstly, individuals hold representations of the *prototypical* attributes of the leader. For instance, a military leader is someone capable of tenacity and fortitude in the face of threat. This holds regardless of which military leader one considers, whether they are one of ours or one of theirs. By contrast, these attributes are not at all representative of religious leaders, who are prototypically reticent and taciturn regardless of which religion we consider. Secondly, a process of *social attraction* results in individuals who demonstrate prototypical attributes being liked more than others who embody these features to a lesser degree. Finally, through a process of *attribution*, prototypical members of the group are accorded higher prestige for who they are, rather than for what they represent for others. This happens regardless of whether the individual themselves value the attributes others enjoy in them. In any case, according to Hogg, leaders inspire followers to the extent that they represent the valued attributes of the group.

In the present study, we extend this focus to our investigation of public opinion concerning the police corps. Clearly, prototypical features are in themselves a social representation of desirable attributes associated with particular roles, not only leadership. One could investigate the prototypical features of leaders, as Hogg (2001) suggests, as well as any other publicly recognised official (e.g. headmaster, police officer, postman, professor, etc.), such that we might be able to supply a list of desirable features associated with any role even if no single individual embodies the list in its entirety (Harré & Sammut, 2013). We can understand, however, that the more a particular individual approximates the full list, the better liked they will be in the public eye. In any such inquiry, those features that emerge as consensually validated within a social group would constitute the prototypical features making up the social representation of the entity involved. Consequently, in the present case, to the extent that real-life police officers approximate these prototypical attributes they stand to be liked, endorsed and respected by the public. On the other hand, to the extent that they do not, they stand to lose popularity and attract resistance in the carrying out of their duties, however legitimate these might be at law. We argue that in fulfilling their policing duties to the satisfaction of a divided public, policing officials need to act in ways that inspire more respect than resistance. Failure to do so risks one side or the other perceiving the police as being on the other side, making the police themselves a target of acrimony. In contrast, to the extent that the police fulfil a groups’ prototypical aspirations of what the police should do in certain situations, they stand to earn protestors’ respect for the boundaries they impose to regulate democratic protest. This is essential in the democratic implementation of rule of law in any country. To this end, the present inquiry was guided by the following research question: *What prototypical features characterise the social representation of policing in Malta?*

## Methodology

The aim of this study was to understand people’s views on policing. The study involved a series of face-to-face, in-depth interviews which were carried out online due to the COVID-19 pandemic.

### Participants

A total of 41 participants were recruited using purposive sampling. Recruiters were employed who passed on details of consenting participants to the researchers. Participants were paid €10.00 for their participation, and recruiters were paid €2.50 per participant who completed the interview. The interviews were conducted with participants from ten different geographic localities across the whole of Malta. Twenty participants were male and twenty-one were female. Ten participants reported a secondary level of education, whilst thirty-one were educated to tertiary level. Eighteen participants reported being in their twenties, nine in their thirties, ten in their forties and four in their fifties. Police officers and their relatives were excluded from participation. Interviewees were all Maltese nationals.

### Interview Topic Guide

The interview topic guide was developed with the aim of understanding people’s views of the police with regards to what the police are *for*. We adopted Buhagiar and Sammut’s (2020) formula for action-oriented social representations inquiry over an object-oriented formulation. The topic guide addressed the public’s views relating to: (a) the role of police officers in society; (b) the (i) justifications for use, and (ii) limits to the authoritative powers of police; (c) the perceived core duties of the police concerning (i) crime prevention, (ii) law enforcement, and (iii) crime resolution; (d) other duties associated with policing, in terms of (i) what the police should help out with, and (ii) what they should refuse to help out with; € the extended duties of the police concerning (i) enforcement, (ii) protection, (iii) public order, (iv) private needs, and (v) quality of life issues; and (f) police functions in (i) surveillance, (ii) stations, and (iii) on the beat.

### Procedure

Prior to starting the interviews, the study was explained to participants over the phone. Participants were made aware of the purpose of the study, completed a demographics sheet and provided explicit verbal/written consent. All participants were informed that the online interviews would be audio-recorded. Each interview session lasted around 45 min. The interviews took place between the 7th May and the 1st June 2020, using Zoom. The time and day of the interview was chosen by the participants. The collected personal details were stored safely, and participant anonymity was safeguarded.

### Data Analysis

All data was analysed using thematic network analysis (Attride-Stirling, 2001). Moreover, the analysis was focused on eliciting both the *consensual* and *controversial* elements of each theme (see below). Data analysis was completed using NVIVO12.

### Thematic Coding

Data was coded at three levels: (a) basic themes; (b) organizing themes; and (c) global themes (Attride-Stirling, 2001). A preliminary coding framework was established, following the coding of one whole transcript, and refined following the further coding of another 6 transcripts. By the 7th transcript, the coding frame was solidified, and general codes started to emerge. The emerging codes were re-visited and collated with every 10th interview that was analysed. This made the dataset manageable and ensured a consistent application of the coding frame. The coding of 41 interviews resulted in a proliferation of codes, which were organised as follows.

### Thematic Structure

A thematic network was derived by establishing (a) basic themes, (b) organizing themes, and (c) global themes, respectively. Firstly, the previously identified codes were grouped into basic themes. *Basic themes* summarised participants’ main points, and incorporated the patterns identified when coding. After consolidation, the dataset yielded 422 codes, which were subsequently grouped into 91 basic themes summarising the dataset. Following this exercise, basic themes revolving around the same topics were gathered together under meaningful *organizing themes*, which were sufficiently distinct from each other. A total of 23 organizing themes emerged from this step. Thirdly, the organising themes were grouped into 3 separate *global themes*: (1) ‘police duties’, (2) ‘public image’, and (3) ‘scope for development’. Furthermore, consensual and controversial data within each global theme was coded separately, in an effort to gain insight into the common-sensical consensus regarding policing in Malta as well as the polemics surrounding the practice. For each global theme, the *consensual* (prototypical) elements constituted notions about which there was no contestation at all (e.g., the view that law enforcement is a police duty). Additionally, the *controversial* elements were also coded. These consisted of notions about which there was contestation, regardless of scale and frequency (e.g., pro vs. contra arguments about body cams). The following section presents these findings in further detail along with illustrative excerpts sourced from the corpus.

## Findings

The social representation of policing in Malta is structured in three organizing themes (Table 1).

**Table 1:** Thematic structure of consensus and controversies

Global theme	Organising theme	Basic theme	Consensus & controversy
Policing	Police duties	Responsibilities	Consensus: To protect; Law enforcement; Investigation; public order
			Controversy: ‘Police should stick to formal duties’ vs ‘Police should go beyond their call of duty’
		Prevention/apprehension	Consensus: prevention & apprehension equally important
			Controversy: ‘Prevention as a priority’ vs ‘Apprehension as a priority’
		Use of force	Consensus: Legitimate if necessary
			Controversy: ‘Used too much’ vs ‘Used too little’
		Random searches	Controversy: ‘Legitimate’ vs ‘not without valid justification’
	Public image	Mentality	Consensus: Not all officers are the same
			Controversy: ‘Mostly positive’ vs ‘Mostly negative’
		Effectiveness	Controversy: ‘Mostly effective’ vs ‘mostly ineffective’
		Trust	Controversy: ‘Mostly trustworthy’ vs ‘not trustworthy’
		Vocational	Consensus: policing is a vocation
		Career	Consensus: policing is undervalued
	Scope for development	Investment	Consensus: Needed in Various Areas
		Police stations	Consensus: Need for well staffed stations
			Controversy: ‘Increase in staff needed’ vs ‘More organisational efficiency needed’
		On the beat	Controversy: ‘More patrols needed’ vs ‘Corruptible’
		Community policing	Consensus: Need to be well equipped and well trained
			Controversy: ‘Builds bridges’ vs ‘overfamiliarity’
		Body cams	Consensus: Serve protection functions for police & citizens alike
			Controversy: ‘Improvement’ vs ‘Privacy issues’
		CCTV	Consensus: Double-Edged sword
			Controversy: ‘Incontrovertible proof’ vs ‘big brother’
		Social media	Controversy: ‘Strengthens communication’ vs ‘frivolous and wasteful’

The first organizing theme pertains to *Police Duties*, constituted by four basic themes. Respondents expressed a general consensus regarding the fact that policing involves the protection of citizens against crime and maintenance of public order, the role of law enforcement, and criminal investigation and prosecution (*Responsibilities*):

*“To maintain public order. To see that everyone follows the rules, both the powerful and the powerless, and that everything works as it should” (Male, 28, Tertiary)*

Respondents also agreed that the police are tasked with general duties to both prevent crime as well as apprehend criminals (*Prevention & Apprehension*):

*“one does not exclude the other. Because, the reality of life is that, however much you try to prevent, there will always be those who do bad things.” (Male, 29, Tertiary)*

*“You prevent by showing people that, if you do bad, …you’re going to pay the price for it.” (Male, 26, Tertriary)*

Finally, respondents agreed that the police carry the authority to use legitimate force if necessary (*Use of Force*). On the other hand, elements within these themes, along with the theme of *Random Searches* proved controversial. Respondents were divided over whether the police should stick to their formal duties or whether policing requires officers to go beyond their call of duty:

*“A police officer shouldn’t have to do everything, there needs to be specialization.” (Male, 55, Tertiary)*

*“A police officer goes to work, but it should also be the case that- … They help whoever needs help, so I do not think it is just a job […] There should be a sense of community as well, in the sense that you help whoever it may be.” (Female, 38, Tertiary)*

Respondents were further divided with regards to whether police duties should err on the side of preventing crime to ensure order, or on the side of apprehending criminals as this amounts to restorative justice and teaches criminals that crime does not pay:

*“You’re going to spend less if you prevent, rather than trying to address the damage after the fact.” (Male, 36, Tertiary)*

*“I’m not sure what they can do exactly to prevent crimes apart from education, but I’m sure some people know that what they’re doing is wrong and still do it anyway” (Female, 22, Tertiary)*

Another point of controversy pertained to whether the police resort to the use of force all too frequently or whether nowadays the police are toothless because their ability to deploy force has been overly restricted. Finally, respondents were divided over whether powers to search citizens at random should be exploited in case they lead to the fortuitous apprehension of criminals or restricted to avoid abuse and a sense of insecurity.

The second organising theme concerned the police’s *Public Image*. A general consensus was expressed by respondents over the fact that not all police are the same and one can find good coppers as well as bad ones (*Mentality*):

*“Not everyone* [is the same]*. Because sometimes, you encounter a police officer who’s all right, and sometimes you meet a police officer and you’re afraid of him, or else you say, ‘I can do anything I want in front of this one, he won’t tell me anything’.” (Female, 25, Tertiary)*

Respondents also agreed that policing is a vocation (*Vocation*):

*“these people are cut out for it, they were sort of born to do it in a sense.” (Male, 23, Tertiary)*

Finally, respondents agreed that careers in the police corps are undervalued (*Career*). The latter two themes did not demonstrate any elements of controversy. By contrast, the *Mentality* theme was also controversial with respect to the fact that, given human diversity in the corps, the mentality of police officers tended generally to be positive, or negative:

*“I feel that the mentality is that, ‘listen, I am here to do my work and do my duty. If a person wants to come, she asked a question, or she is in a particular situation, I will help whenever I can” (Female, 29, Secondary)*

*“I lean more toward the view that, their attitude is that they couldn’t care less.” (Female, 44, Post-Secondary)*

Moreover, two additional themes polarised respondents and did not demonstrate any consensual elements. Respondents were divided over whether the police were generally effective or ineffective in the carrying out of their core duties (*Effectiveness*):

*“professional, helpful, like as a professional I found them, when I presented myself as a professional I found them of a lot of help” (Female, 38, Tertiary)*

*“Because they’re, their sort of obligations aren’t really undertaken well. When they have to investigate I think, they do the bare minimum and don’t really try to find the perpetrator or, and they don’t really try to understand how certain crimes work.” (Female, 22, Tertiary)*

Respondents were also divided over whether, in general, the police were to be regarded as mostly trustworthy or mostly untrustworthy (*Trust*):

*“if I see something or know something which is not one hundred percent right, I have no problem* [in resorting to the police]*” (Male, 46, Tertiary)*

*“I think when you are in a crisis, it is inevitable. You will trust them because you have to.” (Female, 40, Tertiary)*

The final organising theme pertained to *Scope for Development*, where respondents discussed initiatives that could ameliorate policing services. Respondents demonstrated a general consensus that more investment is needed in policing operations across the board (*Investment*). This theme did not demonstrate any controversy:

*“First of all, they should upgrade the police station. I mean this is a place of work where these policemen are coming in everyday. At least make it comfortable, aesthetic for them, I mean it’s degrading for them, for the public as well.” (Male, 46, Tertiary)*

*“Give them* [police] *everything they need to carry out their work…I believe that there is no limit on resources, the more you give them, the more happy people will be at work and the better able they are to do their work.” (Male, 36, Tertiary)*

Respondents further agreed that police stations were important and needed to be well staffed (*Police Stations*). However, respondents were divided over whether police officers should stay put in police stations before responding to citizen requests, or whether they should avoid idle time through increased patrols when possible:

*“We need to increase police in stations. The amount of times that I phoned the police station […] and they told me that they don’t have police to send out is unbelievable.” (Female, 36, Tertiary)*

*“I think it’s more important to have organization and ensure that they are carrying out their duties…organisation does not exist. For example, you enter a police station and see the police chatting away whilst you stand there waiting to talk to someone.” (Male, 55, Tertiary)*

Similarly, respondents agreed that community policing required well prepared officers, who were well-equipped and who demonstrated decent interpersonal skills (*Community Policing*). They disagreed over the fact that community policing can help build bridges with the community or whether it serves as an opportunity for corruption:

*“This* [community policing] *creates a dialogue with the community. They* [police] *are accessible to the citizen, and this is good for the police, so they are approached like friends who can help out in the community.” (Male, 36, Tertiary)*

*“I don’t like it* [community police]*, […] It is going to lead to too much chumminess that I don’t think will be good because then ‘come on, forgive my ticket’”. (Male, 27, Tertiary)*

A similar scepticism was demonstrated in discussing the role of officers on the beat (*On the Beat*). Respondents were divided over whether they saw this as an effective deterrent for crime or whether this gave rise to overfamiliarity with certain members of the community. This theme did not present any consensual elements:

*“If it were up to me it would be* [i.e., they would invest in] *patrolling […] that they see that the law is abided to is the most important thing. That is what teaches people.” (Female, 59, Tertiary)*

*“A police officer, […] can be bribed, there is always going to be that human error of, you know* […] *the institution of law is seemingly infallible, but a person maintaining it can be, can be fallible.” (Male, 26, Tertiary)*

A further cluster of themes concerned the use of technology. Respondents agreed that body-cameras serve protection functions for both officers and citizens alike when incidents arise (*Body Cams*):

*“So, obviously the police will feel protected by having the camera on him, and if he uses force on someone else, he has the evidence as to why he used force.” (Male, 41, Secondary)*

Respondents also agreed that CCTV was a double-edged sword (*CCTV*):

*“I mean, in reality, there are CCTV cameras everywhere, so, I think the police find them useful because they can use them to help them in their investigations […] It does not mean that this is a good thing, because you then have a lack of privacy.” (Female, 20, Post-Secondary)*

On the other hand, both these themes contained elements of controversy. Body cams were held to provide an opportunity for constructive feedback in helping both citizens and police officers understand what might have gone wrong in an incident. Some, however, were concerned that they serve primarily to record citizens in their everyday activities. A similar scepticism was levelled at CCTV. Whilst some argued that it provides incontrovertible evidence, others claimed it provides a Big Brother solution were citizens are unnecessarily monitored in their everyday lives. Finally, the theme of *Social Media* also proved controversial. Some argued that it strengthens police-citizen communication whilst others claimed that it is a frivolous activity as good policing requires a tangible and objective human presence:

*“It helps to have more methods of communication to spread your message. In today’s world, where everything is on social media…it can be very helpful to strengthen communication.” (Female, 29, Secondary)*

*“I find social media to be too virtual - so yes, even though there may be true stories, people can also still write whatever they want […] sometimes social media exaggerates certain things so I think the police needs to be careful from that aspect […] I don’t think that it* [social media] *should be a source* [for policing activity]*” (Male, 36, Tertiary)*

Overall, the prototypical police officer was characterised as one who is responsible for protecting citizens, maintaining public order and enforcing the law. However, it was debated as to whether the police should go beyond the call of duty, focus more on crime prevention as opposed to apprehension, and should be more physically present in the streets. Views related to technological means of surveillance were also divided. Participants disagreed on whether police mistrust is fully merited, or partially unmerited. Moreover, the police force was represented as one requiring improvement, both in terms of policing and public image/communications.

## Discussion

Giddens (1991) has famously noted how no one living in postmodern societies today can follow the precepts of any code without knowing that others living in the same society abide by different, perhaps even incommensurate, precepts. According to Giddens, a key condition of postmodern societies is that of multiple co-existing authorities where the dictates of one are challenged and potentially usurped by another. Scholars as far back as the nineteenth century have warned that such conditions can lead to a state of fragmentation and normlessness that could precipitate the disintegration of social cohesion. Durkheim (1897) famously termed this state of normlessness *anomie*. Contemporary mass protests, such as Black Lives Matter, may arguably be seen as indicative of this state of social fragmentation. In the US, for example, obeying as well disobeying police orders is held by some to involve inherent risks associated with racial profiling and discrimination on the part of the police in general, on behalf of the majoritarian white segment of the American population.

On the other hand, other scholars have defended the collaborative potential that inheres in human nature (see Tomasello & Carpenter, 2007), which enables the formation of self-regulating societies (Sammut, 2018). Asch (1952) notes, for instance, how a society cannot have multiple definitions of crime. To overcome discrepant subjective dispositions, individuals settle their differences by recourse to the thoughts and cognitions of others. This cognitive exercise enables the formation of like-minded coalitions that collectively pursue some mutually acceptable course of action (Buhagiar & Sammut, 2020). In this way, mass protests are catalytic in generating progress through social change (Drury & Stott, 2011; Reicher, 2011). This, however, necessitates competition between diverse coalitions-for-action (Buhagiar & Sammut, 2020; Sammut et al., 2015a, 2015b). For example, the Black Lives Matter marches were countered by the All Lives Matter rallies pursuing an opposite agenda. Similarly, the Brexit referendum in Britain mobilised both ‘for’ and ‘against’ voters who both took to the streets. Consequently, rather than social fragmentation, mass protests may be considered as indicative of healthy societal functioning where democratic freedoms can be openly and publicly expressed.

The human condition of living with diversity thus presents some key challenges in managing politically discrepant perspectives. Sammut et al., (2015a, 2015b) warn that human cognition is biased towards exclusion, which serves to keep traitors who stand to impede coalitional projects at bay. In view of this, Moghaddam’s (2016) call to train citizens in democratic competencies for relating with different others and respecting diversity seems opportune.

Clearly, however, this is no mean feat. The separation of powers along three pillars (legislature, executive, judiciary) that underlies various democratic constitutions worldwide also means that different authorities may help or hinder the achievement of a political cause in different ways. For instance, whilst parliament may force a certain behaviour at law (e.g. social distancing), it is the police who are tasked with enforcement. The extent to which a particular incident constitutes a contravention or otherwise in particular cases is then a matter for the courts to decide. As a result, the achievement of ‘rule of law’ in any society is not only a matter for different institutions working systemically in a coherent way. It is also a matter of the extent to which a citizenry subscribes to upholding the rule of law in everyday life and to avoid civil unrest in trying to redress perceived anomalies (Sammut & Bauer, 2021). An essential element of this behavioural dimension to rule of law is respect towards the authority of legitimate institutions, even when these are the very targets of protest. In practice, this might be highly contentious, as the Black Lives Matter demonstrations have shown. It seems largely self-defeating to submit to police authority when protesting against police brutality. Such circumstances evince the fact that democratic societies, as Sammut (2018) has pointed out, serve merely to kick out incumbents without the need for armed revolt. Democracies offer no guarantee that elected officials will serve selflessly and ethically. Democracy serves merely to settle grievances at the electoral poll.

In such a state of affairs, policing plays a critical role. The police are formally tasked with upholding the rule of law in their routine operations and to prosecute infringements towards the restoration of order. As a result, they control the very boundaries of the expression of democratic rights and freedoms and for this reason are vested with the authority to detain individuals by recourse to the use of force. Consequently, the public’s respect to the authority of the police marks the very cornerstone of civil order and social cohesion. When it fails and the police come to be regarded as yet another self-interested group, public order may be pursued through the formation of bands of vigilantes, armed militias, and ultimately armed conflict. For this reason, we contend that understanding the social representation of policing is a critical scholarly task in the pursuit and implementation of rule of law policy and practice.

Our study sheds light on the identification processes that underlie attitudes of support or disdain towards the police. Respondents in our study generally recognised the authority of the police to maintain law and order, to use force if necessary, and to prevent crime as well as apprehend those who commit it. Respondents also generally agreed that policing is largely undervalued and that it merits increased investment. On the other hand, the issues of trust and effectiveness of police operations polarised respondents. Some expressed a deep scepticism that the police are trusted only out of necessity, that is, because one has to, and that policing is largely ineffective due to the fact that the police do not do the best they can. As a result, they fail to uphold rule of law as well as they should. The consequence of this state of affairs is, as per the proverbial adage: some (literally) get away with murder (and other crimes).

It goes without saying that no institution is operationally effective at all times. The sceptical attitudes unearthed in this study are also countered by positive attitudes towards the police in general. Also, as some respondents conceded, one can find bad apples in any batch. This, however, is more than a moot point. In discussing policing initiatives that are currently being rolled out in Malta to ameliorate the police’s effectiveness along with its public image, such as more officers on the beat as well as community policing initiatives, respondents reaffirmed sceptical attitudes. The root of this scepticism is highly insightful. Respondents remarked that both patrols and community policing afford opportunities for overfamiliarity that may prove to be a breeding ground for corruption. This not only defeats the whole purpose of policing as an activity, it suggests that policing, more than exacerbating problems through inefficiency, can go on to become a societal problem in itself. Corrupt policing not only fails to demarcate criminal from good behaviour, it allows some crime to happen – that lubricated by corruption – whilst blocking the rest. If this were to be genuinely the case, it would be worrisome indeed. What is particularly insightful in our findings, however, is that respondents did not support their worries with real life experiences of witnessing corruption. Rather, they claimed that the police are corruptible by virtue of their human nature.

Respondents understood that the police may be largely effective and well-meaning as an institution, but its officers, as human beings, are subject to the whims of human nature and corruption is, indeed, one such vulnerability. Evolutionary psychologists have long argued that cheating is adaptive and that theory of mind is an evolved cognitive mechanism to detect cheats (Cosmides & Tooby, 2005). In our data, respondents demonstrate what seems to be ‘introspective projection’, suspecting the police to be as human and as corruptible as they themselves might be in similar circumstances and by virtue of this, however well-meaning the institution, the intervention itself is bound to fail. This echoes Nin’s (1961) famous assertion that we do not see things as they are, but as we are. This introspective projection may otherwise counter a positive prototype of the police that serves social identification processes. If we need officers who are committed to their civic role, approachable in times of need, as well upright and non-corruptible, as the prototypical features evincing from our investigation suggest, we might need to look beyond human nature.

 In this respect, the present study unearths a seemingly apposite remedy. Respondents demonstrated sceptical attitudes towards technological policing systems, namely body-cams and CCTV. They claimed that these systems are also subject to misuse, such as unwarranted surveillance and violations of privacy. In a sense, they were wary of whose eyes lurk behind the screen – assuming once again a corruptible and potentially malicious perpetrator gaining access to their data. One could also argue that such technological systems are resisted in light of the awareness that one’s own misdeeds will not go unnoticed. This again constitutes introspective projection as in the previous case. However, respondents also conceded that technological systems, body-cams in particular, offer an opportunity for constructive feedback when analysing incidents. They claimed that body-cams offer protection against abuse to the police and citizens alike, given that abuse can arise from either source – as both are equally human. This suggests that inter-objective technological systems (Latour, 1996; Sammut et al., 2013) may overcome human deficiencies by relegating the act of scrutiny against corrupt misuse to technological artefacts that offer a satisfactory compromise to interacting – suspect as well as suspicious – parties.

## Conclusion

The present study has investigated the social representation of policing in Malta. Policing was demonstrated to involve various core duties associated with respecting and protecting law and order as well as the apprehension of criminals. A general consensus emerged regarding the need for wider investment. Yet, the public image of the police emerged as a contested issue, partly due to the fact that as human beings, police officers are held to be potentially corruptible. We have argued that this constitutes introspective projection on the part of lay citizens who identify vulnerabilities in the police similar to their own, rooted in human nature. We claim that this psychological mechanism may impede positive identification with the police in certain cases, given they cannot realistically match the prototype of the approachable, competent as well as incorruptible officer. Consequently, the public image of the police is necessarily fraught with tension concerning the extent to which they can be trusted. In this light, contemporary efforts at increasing trust in the police as measured in public opinion polls may prove to be a mere red herring. We suggest that the incorporation of technological systems in the execution of policing duties, such as body-cams, might offer a more effective reassurance measure that satisfies the police and citizens alike. We conclude by arguing that such technological developments are necessary today more than ever if the police are to be trusted enough to garner the respect of citizens even when the latter manifestly take issue with governing authorities and public institutions in line with highly valued democratic ideals.
